# 

**Published:** 1886-10

**Authors:** 


### BOOKS AND PAMPHLETS RECEIVED.

OSTEOLOGY OF CONURUS CAROLINENSIS AND A NAVAJO SKULL. By R. W. Shufeldt, M. D., U. S. A.

JARESBERICHT DER K. CENTRAL THIERARZNIE-SCHULE IN MUNCHEN, 1884–85. Leipzig, 1886.

SECOND ANNUAL REPORT OF THE BUREAU OF ANIMAL INDUSTRY FOR THE YEAR 1885. Washington, 1886.

AMERICAN VETERINARY COLLEGE. Twelfth Annual Announcement. Session, 1885–86.

REPORT OF THE THIRD ANNUAL MEETING OF THE NATIONAL VETERINARY MEDICAL ASSOCIATION, held in Washington, December 1885.

ICHTHYOL UND RESORCIN ALS REPRASENTANTEN DER GUIPPE REDUZIERENDER HEILMITTEL VON DR. P. G. UNNA. Hamburg & Leipsig, 1886.

DETROIT COLLEGE OF MEDICINE. Announcement for session, 1886–7.

HOMOEOPATHY AS VIEWED BY A MEMBER OF THE MASSACHUSETTS MEDICAL SOCIETY. By Vincent Y. Bowditch, A. B., M. D.

ADDRESS DELIVERED BEFORE THE ALUMNI ASSOCIATION OF THE DEPARTMENT OF MEDICINE AND SURGERY OF THE UNIVERSITY OF MICHIGAN. By Charles J. Lundy, M. D. Ann Arbor, 1886.

THE MEDICAL REGISTER OF NEW YORK, NEW JERSEY AND CONNECTICUT, for the year commencing June 1, 1886. William P. White, M. D., Editor. New York, G. P. Putnam's Sons, 1886.

TYROTOXICON ; its presence in poisonous ice cream ; its development in milk ; and its probable relation to cholera infantum and kindred diseases. By Victor C. Vaughn, M. D.

SOME RECENT EXPERIENCES IN CHEMICAL SURGERY. By Donald Maclean, M. D.

GALVANO-CAUTERY IN DISEASES OF THE PROSTATE BLADDER AND URETHRA. By Robert Newman, M. D.

SURGICAL NOTES FROM THE BOOK OF A GENERAL PRACTITIONER. By William C. Wile, M. D., Newtown, Conn.

RHEUMATOID OSTEOARTHRITIS. By Alexander Hadden, M. D., New York.

THE PAROCCIPITAL, A NEWLY RECOGNIZED FISSURAL INTEGER. By Burt G. Wilder, M. D.

STUDIES ON THE CONTAGIOUS DISEASES OF INSECTS. By S. A. Forbes—Bulletin of the Illinois State Laboratory, Art. IV., Vol. II.

### LIST OF CONTRIBUTORS.

Frank S. Billings, D. V. S.
Richard W. Burke, M. R. C. V. S.
A. W. Clement, V. S.
Edward K. Dunham, M. D.
H. F. James, V. S.
James Law, F. R. C. V. S.
John C. Peters, M. D.
Thomas B. Rogers, D. V. S.
Prof. Schutz.
R. W. Shufeldt, M. D.
Edward C. Spitzka, M. D.
G. Archie Stockwell, M. D., F. Z. S.
John Bland Sutton, M. D., F. R. C. V. S.
James A. Waugh, V. S.
A. Stickes.
Thomas Walley, M. R. C. V. S.

### INDEX TO VOLUME VII.

PAGE
American Veterinary College Commencement . . . . . . . . . . . 235
Anatomy of the Dolphin's Brain, Notes on . . . . . . . . . . . 224
Animals, Diseases of the Circulatory Organs in . . . . . . . . . . 323
Relapsing Fever in. . . . . . . . . . . . . . . . . . . . 283
Effects of Lightning, Fire, Smoke and Steam upon. . . . 267
Armadillo, Rectum of . . . . . . . . . . . . . . . . . . . . . 237
Autopsies at Central Park Menagerie . . . . . . . . . . . . . . 233
Avian Tuberculosis, or Amœbic Warfare . . . . . . . . . . . . 329
Azoturia . . . . . . . . . . . . . . . . . . . . . . . . . . 139
Barsati, or Atrophic Cancer . . . . . . . . . . . . . . . . . . 368
BILLINGS, F. S., in Paris . . . . . . . . . . . . . . . . . . . 147
Rabies in the Dog in its Relation to Man . . . . . 161
Tuberculosis with Reference to Phthisis Pulmon . . 62
Blood, Coagulation of the . . . . . . . . . . . . . . . . . . . 318
Books and Pamphlets Received . . . . . . . . . . . 160, 240, 328, 421
Bromo Soda . . . . . . . . . . . . . . . . . . . . . . . . . 400
BURKE, R. W., Azoturia more Especially with Reference to its Nomenclature and pathology . . . . . . . . . 139
Barsati, or Atrophic Cancer . . . . . . . . . . . 368
Intermittent Diarrhœa in Camels . . . . . . . . . 241
Camels, Febrile Intermittent Diarrhœa in . . . . . . . . . . . . 241
Case Department . . . . . . . . . . . . . . . . . . . . . . 233, 400
Cat, Cocaine Poisoning in a . . . . . . . . . . . . . . . . . . 236
Cattle, Diagnosis of Tuberculosis in . . . . . . . . . . . . . . 291
CLEMENT, A. W., Three Cases of Sarcoma in the Horse . . . . . . 400
Comparative Anatomy of the Pyramid Tract . . . . . . . . . . . 1
Contagious Diseases, Immunity from . . . . . . . . . . . . . . 237
Defects of the Present U. S. Army Veterinary System . . . . . . . 399
Dogs, Research into the Pathology of the Diseases in . . . . . . . . 322
Tuberculosis in a . . . . . . . . . . . . . . . . . . . . 232
DUNHAM, E. K., Autopsy of Pig-tailed Monkey . . . . . . . . . . 233
Editorial Department . . . . . . . . . . . . . . . . . 147, 224, 289, 394
Elephant's Brain . . . . . . . . . . . . . . . . . . . . . . . 398
Errata . . . . . . . . . . . . . . . . . . . . . . . . . . . 150
FLEMING, GEORGE, F. R. C. V. S. . . . . . . . . . . . . . . . . 143
Glanders and Pleuro-Pneumonia in Russia . . . . . . . . . . . . 232
The Differential Patho-Anatomical Diagnosis in . . . . . 196
Horse, Castration of the . . . . . . . . . . . . . . . . . . . . 230
Tuberculosis in the . . . . . . . . . . . . . . . . . . . 230
Hydrophobia. . . . . . . . . . . . . . . . . . . . . . . . . . 412
Dr. Spitzka on. . . . . . . . . . . . . . . . . . . . 289
How can we Prevent False . . . . . . . . . . . . . . 244

### Index.

PAGE
JAMES, H. F., Osteo-Porosis . . . . . . . . . . . . . . . . . . 193
Lambs, Nephritis in . . . . . . . . . . . . . . . . . . . . . . . . 324
LAW, JAMES, The Role of Microbes and Their Chemical Products . 360
Medico-Legal Society, Meeting of . . . . . . . . . . . . . . . . . 293
Microbes, The Role of . . . . . . . . . . . . . . . . . . . . . . . 360
Milk, From a Medico-Sanitary Standpoint . . . . . . . . . . . . . 101
Monkey, Pig-tailed, Autopsy of . . . . . . . . . . . . . . . . . . 233
Montreal Veterinary College, Commencement of . . . . . . . . . . 316
National Veterinary Medical Association, . . . . . . . . . . . . 151, 406
Association, Great Britain . . . . . . . . . . 407
Nature's Repairs to Beaks of Birds . . . . . . . . . . . . . . . . 357
New Jersey State Veterinary Society . . . . . . . . . . . 156, 309, 410
New York College of Veterinary Surgeons, Commencement . . . . 236
State Academy of Veterinary Science . . . . . . . . . 314
Obituary . . . . . . . . . . . . . . . . . . . . . . . . . . . 158, 237
Osteo-Porosis . . . . . . . . . . . . . . . . . . . . . . . . . . . 193
Ozone and Pneumonia . . . . . . . . . . . . . . . . . . . . . . . 290
Pasteur and his Work . . . . . . . . . . . . . . . . . . . . . . . 190
Pasteuriana . . . . . . . . . . . . . . . . . . . . . . . . . . . . 412
PETERS, J. C., Relapsing Fever in Animals . . . . . . . . . . . . 283
Phenomena and Evidence of Serpent Poison . . . . . . . . . . . . 377
Pleuro-Pneumonia, Contagious . . . . . . . . . . . . . . . . . . 396
Proceedings Veterinary Medical Societies . . . . . . 151, 235, 293, 405
Progress of Veterinary Science . . . . . . . . . . . . . . 236, 318, 411
Pure Pepsin . . . . . . . . . . . . . . . . . . . . . . . . . . . . 411
Putnam Nail Company, The . . . . . . . . . . . . . . . . . . . . 149
Pyramid Tract, The . . . . . . . . . . . . . . . . . . . . . . . . 148
Rabies . . . . . . . . . . . . . . . . . . . . . . . . . . . . . . . 229
In the Dog and its Relation to Man . . . . . . . . . . . . . . 161
Reviews . . . . . . . . . . . . . . . . . . . . . . . . . 159, 238, 325, 414
ROGERS, T. B., Milk from a Medico-Sanitary Standpoint . . . . . . 101
Sarcoma in the Horse . . . . . . . . . . . . . . . . . . . . . . . 400
SCHUTZ, PROF., Diagnosis in Glanders . . . . . . . . . . . . . . 196
Science and the State . . . . . . . . . . . . . . . . . . . . . . . 229
SHUFELDT, R. W., Nature's Repairs to Beaks of Birds . . . . . . . 357
SPITZKA, E. C., Comparative Anatomy of the Pyramid Tract . . . . 1
How can we Prevent False Hydrophobia . . . . . 244
STOCKWELL, G. A., Phenomena of Serpent Poison . . . . . . . . . . 377
SUTTON, J. B., Avian Tuberculosis . . . . . . . . . . . . . . . . 329
Typhoid Fever in Animals . . . . . . . . . . . . 218
Tendons, Research into the Union of . . . . . . . . . . . . . . . 318
Testimonial . . . . . . . . . . . . . . . . . . . . . . . . . . . . 150
Thyroid Gland . . . . . . . . . . . . . . . . . . . . . . . . . . . 319
Tiger, Autopsy of . . . . . . . . . . . . . . . . . . . . . . . . . 234
Trichiniasis in Prussia . . . . . . . . . . . . . . . . . . . . . . 230
Tuberculosis with Reference to Phthisis . . . . . . . . . . . . . 62
Typhoid Fever in Animals . . . . . . . . . . . . . . . . . . . . . 218
United States Veterinary Medical Association . . . . . . . . . . 405
United States Army Veterinarians . . . . . . . . . . . . . . . . . 390
Venereal Diseases in the Lower Animals . . . . . . . . . . . . . 212
Veterinary Legislation . . . . . . . . . . . . . . . . . . . . . . 291
Medicine in Prussia . . . . . . . . . . . . . . . . . 236
Vulva, New Constrictor for . . . . . . . . . . . . . . . . . . . . 412
WALLEY, THOMAS, M. R. C. V. S. . . . . . . . . . . . . . . . . . 386
The Effects of Lightning on Animals . . . . . 267
Venereal Diseases in the Lower Animals . . . . 212
WAUGH, J. A., Army Veterinarians . . . . . . . . . . . . . . . . 390

